# Multi-Sensor Perception Strategy to Enhance Autonomy of Robotic Operation for Uncertain Peg-in-Hole Task

**DOI:** 10.3390/s21113818

**Published:** 2021-05-31

**Authors:** Li Qin, Hongyu Wang, Yazhou Yuan, Shufan Qin

**Affiliations:** School of Electrical Engineering, Yanshan University, Qinhuangdao 066012, China; 972472486@stumail.ysu.edu.cn (H.W.); yzyuan@ysu.edu.cn (Y.Y.); 1736941343@stumail.ysu.edu.cn (S.Q.)

**Keywords:** robot, multi-sensor perception, Bayesian probability, fusion decision, autonomy operation

## Abstract

The peg-in-hole task with object feature uncertain is a typical case of robotic operation in the real-world unstructured environment. It is nontrivial to realize object perception and operational decisions autonomously, under the usual visual occlusion and real-time constraints of such tasks. In this paper, a Bayesian networks-based strategy is presented in order to seamlessly combine multiple heterogeneous senses data like humans. In the proposed strategy, an interactive exploration method implemented by hybrid Monte Carlo sampling algorithms and particle filtering is designed to identify the features’ estimated starting value, and the memory adjustment method and the inertial thinking method are introduced to correct the target position and shape features of the object respectively. Based on the Dempster–Shafer evidence theory (D-S theory), a fusion decision strategy is designed using probabilistic models of forces and positions, which guided the robot motion after each acquisition of the estimated features of the object. It also enables the robot to judge whether the desired operation target is achieved or the feature estimate needs to be updated. Meanwhile, the pliability model is introduced into repeatedly perform exploration, planning and execution steps to reduce interaction forces, the number of exploration. The effectiveness of the strategy is validated in simulations and in a physical robot task.

## 1. Introduction

Several recent studies have demonstrated that robotic operations may no longer be targeted at specific objects and structured tasks. For example, in the medical field, robots autonomously perform large-scale pharyngeal swab sampling to reduce the risk of COVID-19 (Coronavirus Disease 2019) in health care workers [1]. In industry, robots can replace humans in the autonomous assembly of circuit breaker components and ensure a compact assembly [2]. Similar robotic operations can also extend to the robotic refueling and charging of vehicles. A critical step that exists in the above tasks is the robotic peg-in-hole operation with an uncertain object. This type of operation usually has to meet force and position accuracy requirements under the constraints of narrow spaces and uncertain objects. It is essential to obtain feature information of uncertain objects for the performance of frequent interaction operational tasks in narrow spaces.

Visual sensors are considered to be the most common and direct method, which are used to perceive important features of the uncertain object. In [3,4] they perceived the hole posture by a single vision or multi-level vision for axial hole assembly and rivet-in-hole Insertion. However, narrow uncertain objects often cause visual occlusion. For example, during refueling for public service, the end of the fuel gun is not available to the tank well through the vision sensor under the condition of uncertain object features, due to the physical occlusion of the robot itself and the tank housing. Occlusion can lead to sensors not being able to acquire the needed information [5] and force task termination. To solve this problem, some methods have been proposed, such as introducing a heuristic approach [6], which compensates for the residual visual information by a priori knowledge. Nevertheless, the process of demonstration and retrieving data is difficult to experiment in the real world unstructured environment, such as space, deep sea and disaster scene. For frequent interaction operational tasks, tactile sensors can provide richer local information than visual sensors, such as shape, position and friction. Many researches are concerned with predicting grasp stability [7] or re-grasp [8] by the information from tactile feedback. Others use tactile sensors to estimate target position [9] and local surface texture [10,11]. Although the tactile sensor avoids to some extent the shortcomings of the visual absence, it is dependent on the contact state between the robot and operating object. In other words, tactile sensors cannot provide the necessary information when the robot is not in contact with the operating object. Humans instinctively have the ability to seamlessly combine the visual and tactile senses to perceive their surroundings accurately and evaluate the operations being performed [12]. Vision sensors can provide global information for accurate reach. Tactile sensors can estimate local information during operation in the presence of visual errors and missing data due to occlusion [10]. It is a feasible solution idea to achieve the complementarity and concurrency of these two sensors in the robotic autonomy operation.

Some methods based on various learning algorithms that fuse visual and tactile information are proposed, for example, in-depth researches on grasping stability prediction [13,14] and shape estimation [15]. When operating in narrow spaces, Lv et al. [16] fused visual and tactile information using the SVM (support vector machine) algorithm, which enabled the robot to open the cover and insert the charging plug into the charging port autonomously. Shaidah Jusoh et al. [17] proposed a multimodal information fusion method for robots to recognize actions and generate tasks in industrial assembly environments. Nevertheless, multimodal information fusion requires prior knowledge of the task to obtain realistic performance. When this prior knowledge is not available, in [18], a multi-modal representation learning approach with self-supervised functionality, which incorporated visual, force, and robot motion information, was employed to complement the absence caused by visual occlusion in a frequent interaction task. However, the implementation of these methods, whether offline or online learning, requires a large amount of data as the basis. Therefore, these methods are not applicable to operating objects where it is difficult to obtain feature datasets for uncertain objects in advance.

Compared to the above methods, the approach based on Bayesian probabilistic techniques has a clear advantage that a large amount of training data is not required in fusing multi-sensor information. The results in [19] also demonstrated that approaches based on Bayesian probabilistic techniques are significantly superior to neural network-based approaches. In [20], a Bayesian filtering framework was proposed to fuse the residual visual information with the tactile information from the GelSight contact sensor to track the objects operated by the robot in hand. Although using tactile sensors are an effective solution to the problem of visual occlusion, it has high requirements for the working environment as a precision instrument. For special operating environments or special tasks, it may be inconvenient to install dedicated tactile sensors or impossible to collect information. In order to reduce the reliance on dedicated tactile sensors, they are replaced by force/torque sensors. For instance, Fan Zhang et al. [21] used a probabilistic tracking method using a Bayesian network that integrated multimodal information (force information and position information) to estimate the posture of the human body in real-time to dress for disabled people without a camera, even when the person has a sudden unexpected movement. However, a pre-constructed object model is required, which is difficult for the previously mentioned operating object. In the field of robotic assembly, Korbinian et al. [22] presented a framework for tracking visual and tactile information assembly to perform assembly operations for multiple hole types. Besides, some researchers have fused haptic and visual information using a Bayesian framework to achieve the estimation of target positions of assembled objects in industrial assembly [23,24]. In addition to estimating the target position, the estimation of shape and other features and the decision capability, which empowers the robot to evaluate the quality of the task (how well the task was completed), can further improve autonomy.

**Figure 1:**
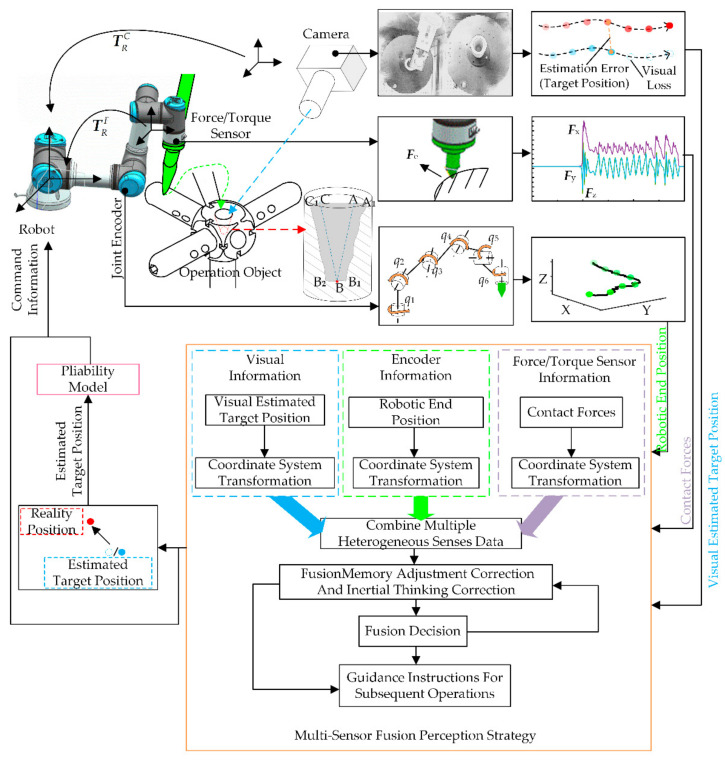
The solution scheme for robotic operating.

To achieve robotic autonomy operation without human intervention and without making any provisions for an uncertain object, the designed solution scheme in this paper is shown in Figure 1. Considered the possibility of visual deficits, contact force information from the force/torque sensor and position information from the robot joint encoder are introduced. The information from the three sensors is used in a multi-sensor perception strategy (MSP) to obtain accurate features of the operating object and guide the operation. The strategy performs an exploration–correction–decision–correction process, as shown in the orange box in Figure 1. In the strategy, the interactive exploration method (IE) is first presented to obtain the features of the uncertain object without a priori knowledge. It integrates the Bayesian posterior probabilities obtained from multimodal information (visual, contact force and position) to features estimated starting value, i.e., the target position and shape of the uncertain object. Then, considered that the dynamic interaction process may increase the uncertainty of the operating object, memory adjustment and inertial thinking are introduced as correction methods to improve the accuracy of the estimates further.

To achieve the function of the robot deciding the next operation by relying on its own judgement, a fusion decision strategy (FD) is designed which is adapted to fine-grained operations with multiple requirements, such as operating accuracy and preload. The purpose of this is to avoid the errors that can arise from single-evidence decisions, for example, when evidenced by position only, substandard quality of operation may occur (i.e., the operating tool reaches the target position but is not installed solidly in the peg-in-hole task). When evidence by contact force only, it can be incorrectly judged as satisfying the task requirements, because an interaction during operation creates a contact force similar to the expected one. The fusion decision strategy is used after each exploration of operating object features. Unlike existed research works that only used position accuracy as an indicator, the designed fusion decision strategy fuses both position and force evidence information in the D-S theoretical framework and includes their uncertainties to guide the subsequent operations.

Finally, command information, such as the features of the uncertain object and the evaluation results of the operation, from the MSP is sent to the robot to guide the operation. As shown in the pink box in Figure 1, the flexibility model is introduced to further optimize the robotic interaction behavior with the operating object.

This paper includes the following contents. In Section 2, There are major problems that robots need to face for autonomous operation under uncertain objects. In Section 3, the proposed multi-sensor perception strategy is presented in detail. In Section 4, the control system is proposed. In Section 5, simulations based on the robot model established are performed to verify the feasibility of the proposed method and provided parameter references for the experiments. Then, physical robot operation experiments with dynamic objects are conducted to further verify the effectiveness of the proposed method faced with more uncertain objects. In Section 6, conclusions are given.

## 2. Problem Description

Based on the above analysis, the following issues will be investigated in this paper:

(1) how to perceive the features of the uncertain operating object,
(2) how to achieve make an automatic decision for the robotic operation,
(3) how to achieve safety interaction between the robot and the uncertain operating object.

For the critical step of the pen-in-hole task, a mathematical description of the above problem was presented. As shown in Figure 1, an internal equivalent model of the operation object is drawn on the right side. The cross-section where the robot interacts with the uncertain operation object is extracted to establish an equivalent model of the uncertain operation object. The inner shape and the target position are selected as the features of the uncertain operation object. It is worth noting that the shape mentioned here is the overall shape of the proposed uncertain operation object. The irregular interior surface has no significant effect on the features to be perceived, so it can be ignored. In addition, for perception algorithms, since the robotic end is simplified to a point with no width and the inner wall is inclined, the uncertain operating object is simplified to a triangle, as shown in Figure 1. Finally, the shape and position can be expressed by three vertices:

(1) $$X_{d}=\left[\begin{array}{ccc} P^{a} & P^{b} & P^{c} \end{array}\right]$$

where $P^{a}\in {\mathbb{R}}^{D}$, $P^{b}\in {\mathbb{R}}^{D}$, $P^{c}\in {\mathbb{R}}^{D}$ are the positions of vertex A, vertex B and vertex C of the triangle, respectively. D denotes the space dimension where the robot is located. The position $P^{b}$ of vertex B is utilized to represent the target position $X_{e}$ of the uncertain operating object.

Besides visual error and occlusion, the operating object may generate continuous dynamic effects due to the interaction process, which can increase uncertainty. The uncertainty can be expressed as $\omega_{e}$. The estimated position of the uncertain object by vision sensors can be expressed as:

(2) $$X_{e,\mathrm{vision}}=X_{e,\mathrm{reality}}+\omega_{e}$$

where $\omega_{e}$ contains visual errors, which are usually non-linear and uncertain. It is also very expensive to recognize and predict for $\omega_{e}$. Therefore, the target position of the uncertain operating object $X_{e}$ can be obtained by estimating the local features of the uncertain operating object $X_{d}$. When the estimate is accurate enough, $X_{e}$ is $X_{e,\mathrm{reality}}$.

Moreover, in practical application, the operation task performed by the robot is usually composed of several continuous nail hole operations. Each contact behavior induces dynamic interactions at the operational object connections, resulting in coupling effects of robot operational errors and system stability. This coupling effect accumulates and increases over the course of the task. From the above description and analysis, it is clear that for each peg-in-hole operation, the robot must perform with high operational accuracy and apply appropriate forces to the operational object during continuous and large task volumes. That is, the contact force needs to satisfy:

(3) $$F_{e}\in \left(F_{\mathrm{preload}},F_{\max}\right)$$

where $F_{e}$ is the contact force at the robotic end, $F_{\mathrm{preload}}$ is the preload force in accordance with the operational requirements, $F_{\max}$ is the maximum contact force allowed. On the one hand, a very high force is not allowed due to the safety of the whole system. On the other hand, a tiny force is also not qualified because there should be enough preload to ensure that each step of the operation is stable, ensuring that the whole structure is stable and reliable [25].

If the robot can make timely autonomy decisions based on the collected limited information to guide the subsequent movements, the number of contacts can be effectively reduced and thus the dynamic effects are reduced. In addition, it also reduces human involvement, which effectively avoids human errors and workload. Therefore, a variable ξ, which represents the result of the decision, is needed to evaluate the quality of the task and guide subsequent operations.

## 3. Design of Multi-Sensor Perception Strategy

**Figure 2:**
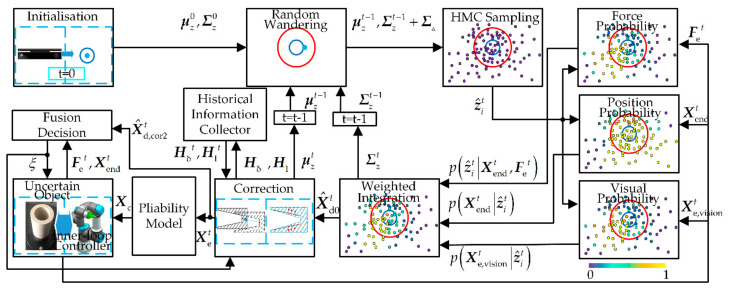
Multi-sensor perception strategy.

The multi-sensor perception strategy is divided into two parts, i.e., interactive exploration and fusion decision, which are shown in Figure 2. Firstly, in the interactive exploration part, the information from the three sensors is seamlessly combined to obtain features estimated starting value ${\hat{X}}_{d0}^{t}$. The correction component is used to adjust the ${\hat{X}}_{d0}^{t}$, which will obtain accurate operating object features ${\hat{X}}_{d,\mathrm{cor}2}^{t}$. Then, the information in ${\hat{X}}_{d,\mathrm{cor}2}^{t}$ is sent to the fusion decision part and the pliability model part, respectively. The fusion decision part will provide the robot controller and exploration part with ξ to guide subsequent movements. Finally, ξ and the commanded position $X_{c}$ provided by the pliable model will together guide the subsequent robot movements.

### 3.1. Interactive Exploration

In the case of imperfect vision sensors, the features of the uncertain operating object are explored by integrating three sources of information. Given the position of the robotic end (i.e., $X_{\mathrm{end}}^{t}$ in (3.1.3)), the force information between the robot and the uncertain operating object (i.e., $F_{e}{}^{t}$ in (3.1.4)) and estimated operating object features by vision (i.e., $X_{e,\mathrm{vision}}^{t}$ in (3.1.5)), particles (each particle ${\hat{z}}_{i}^{t}$ corresponds to a set of features of the uncertain operating object $X_{d}$) are weighted integrated to estimate the features of the uncertain operating object. The coordinate transformation between the three sensors can be achieved by the transformation matrix $T_{R}^{C}$ and $T_{R}^{F}$. Furthermore, memory adjustment and inertial thinking methods are introduced to correct the shape feature and target position feature of the uncertain operating object, respectively, to improve the estimation accuracy.

#### 3.1.1. Initialization

It is assumed that the features of the uncertain operating object at each moment obey the Gaussian distribution and include the dynamics of operating object prediction.

(4) $$P=N(\mu_{z}^{t},\Sigma_{z}^{t})$$

where $\mu_{z}^{t}$ represents the mean vector of the Gaussian distribution, $\Sigma_{z}^{t}$ represents the covariance matrix of Gaussian distribution. $\mu_{z}^{t}$ is the features of the uncertain operating object at the current moment. For initialization, $\mu_{z}^{0}$ is the initial features of the uncertain operating object collected by the depth camera and $\Sigma_{z}^{0}$ is the error of the depth camera. When t>0, the definition of $\mu_{z}^{t}$ and $\Sigma_{z}^{t}$ will be introduced in detail in Section 3.1.6.

#### 3.1.2. Random Wandering of Sampled Particles

The particle random walk is realized by adding a constant diagonal matrix to the covariance matrix of the Gaussian distribution. The purpose is to introduce dynamic effects in the uncertain operating object between two moments in the model. Hybrid Monte Carlo sampling (HMC sampling) according to the new Gaussian distribution is performed to obtain the particles:

(5) $${\hat{Z}}^{t}={\left[{\hat{z}}_{1}^{t},{\hat{z}}_{2}^{t},\cdots ,{\hat{z}}_{N}^{t}\right]}^{T}$$

where t is the current moment and N is the number of sampled particles. ${\hat{Z}}_{i}^{t}$ is a six-dimensional vector due to the representation of $X_{d}$.

The random walk of particles is defined as:

(6) $$p\left({\hat{z}}_{i}^{t}\right)=N\left({\hat{z}}_{i}^{t}\left|\mu_{z}^{t-1},\Sigma_{z}^{t-1}+\Sigma_{△}\right.\right)$$

where $\Sigma_{△}$ is a constant diagonal matrix, which contains the dynamics of the predicted uncertain operating object between two moments.

In the following section, based on the position information and force information of the robotic end and visual estimated operating object features at the current moment, probability calculations are performed on each sampled particle to recursively design a Gaussian distribution so that the features of the uncertain operating object at each moment can be estimated.

#### 3.1.3. Estimating the Probability of the Features of the Uncertain Object Based on Position Information

**Figure 3:**
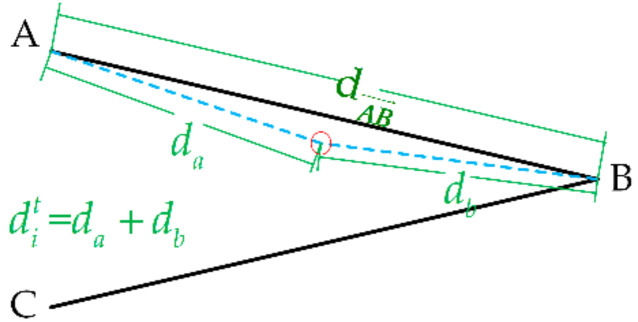
Probabilistic model based on the position information. The solid black line indicates the uncertain operating object and the red circle indicates the end of the robot.

The robotic end position $X_{\mathrm{end}}^{t}$ is used to estimate the probability of the features of the uncertain operating object, as shown in Figure 3. $d_{i}^{t}$ is defined as the distance between the position of the current robotic end and the estimated uncertain operating object ${\hat{Z}}_{i}^{t}$. Each $d_{i}^{t}$ is obtained by calculating $d_{i,\mathrm{up}}^{t}$ and $d_{i,\mathrm{down}}^{t}$, where $d_{i,\mathrm{up}}^{t}$ is the sum of $d_{a}$ and $d_{b}$ and $d_{i,\mathrm{down}}^{t}$ is the sum of $d_{c}$ and $d_{b}$, respectively. Among them, $d_{a}$ is the distance between the robotic end and the vertex A, $d_{b}$ is the distance between the robotic end and the vertex B, $d_{c}$ is the distance between the robotic end and the vertex C. Note that whether $d_{i,\mathrm{up}}^{t}$ or $d_{i,\mathrm{down}}^{t}$ is used depends on the current position of the robotic end in the uncertain operating object. In other words, if the end of the robot collides with $\vec{AB}$, $d_{i,\mathrm{up}}^{t}$ is used, otherwise, $d_{i,\mathrm{down}}^{t}$ is used. Assumed that the robotic end is on the boundary (i.e., $d_{i}^{t}{=d}_{\vec{AB}}$ or $d_{i}^{t}{=d}_{\vec{CB}}$) when the robot happens to collide with the uncertain operating object. Therefore, under the conditions of a given uncertain operating object, the probability of the current robot end position colliding with the uncertain operating object can be expressed as:

(7) $$p\left(X_{\mathrm{end}}^{t}\left|{\hat{z}}_{i}^{t}\right.\right)=N(d_{i}^{t}\left|\mu_{d}\right.,\sigma_{d}^{2})$$

where $\sigma_{d}^{2}$ is the covariance matrix of the Gaussian distribution function, $\mu_{d}$ can be defined as:

(8) $$\mu_{d}=d_{o}=\left\{\begin{array}{c} d_{\vec{AB}},\mathrm{the}\;\mathrm{end}\;\mathrm{of}\;\mathrm{the}\;\mathrm{robot}\;\mathrm{collides}\;\mathrm{with}\;\vec{AB}\\ d_{\vec{CB}},\mathrm{the}\;\mathrm{end}\;\mathrm{of}\;\mathrm{the}\;\mathrm{robot}\;\mathrm{collides}\;\mathrm{with}\;\vec{CB} \end{array}\right.$$

where $d_{o}$ is the mean of the Gaussian distribution. When the end of the robot collides with $\vec{AB}$, the mean is set as $d_{o}{=d}_{\vec{AB}}$. When the end of the robot collides with $\vec{CB}$, the mean is set as $d_{o}{=d}_{\vec{CB}}$.

#### 3.1.4. Estimating the Probability of the Features of the Uncertain Object Based on Force Information

The contact force $F_{e}{}^{t}$ of the robotic end collected by the six-dimensional force sensor (by the contact model in the simulation) is used to calculate the probability of each particle. The normal collision position ${\hat{n}}_{i}^{t}$ is obtained based on each particle ${\hat{Z}}_{i}^{t}$ corresponding to the uncertain operating object and the current position of the robotic end $X_{\mathrm{end}}^{t}$. The friction cone ${\hat{c}}_{i}^{t}$ is defined with the normal ${\hat{n}}_{i}^{t}$ as the axis. 

**Figure 4:**
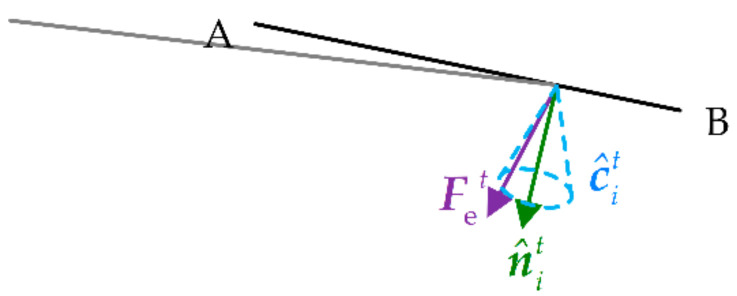
Probabilistic model based on force information. The solid black line indicates the uncertain operating object and the solid gray line indicates the end of the robot.

When the direction of the end contact force is inside the friction cone, the particle corresponds to the uncertain operating object with the highest probability, as shown in Figure 4. In other words, the angle $\theta_{i}^{t}$ between the end contact force $F_{e}{}^{t}$ and the normal ${\hat{n}}^{t}{}_{i}$ should be less than the angle $\theta_{f}^{}$ of the defined friction cone. The probability model based on force information can be defined as follows. When the end contact force $F_{e}{}^{t}$ is inside the friction cone ${\hat{c}}_{i}^{t}$, the probability of the particle ${\hat{Z}}_{i}^{t}$ is defined as 1. When the end contact force $F_{e}{}^{t}$ is outside the friction cone ${\hat{c}}_{i}^{t}$, the probability of the particle ${\hat{Z}}_{i}^{t}$ is defined as a Gaussian distribution function. Therefore, the probability of the force for each particle sampled is defined as:

(9) $$p\left(F_{e}{}^{t}\left|{\hat{z}}_{i}^{t}\right.\right)=\left\{\begin{array}{cc} 1, & \theta_{i}^{t}\leq \theta_{f}\\ N\left(\theta_{i}^{t}\left|\theta_{f}^{}\right.,\sigma_{f}^{2}\right), & \theta_{i}^{t}{>\theta }_{f}^{} \end{array}\right.$$

where $\sigma_{f}^{2}$ is the covariance matrix of the Gaussian distribution function.

#### 3.1.5. Estimating the Probability of the Features of the Uncertain Object Based on Visual Information

When the robot starts to move toward the operation object, vision can easily provide feature information of the object. The current moment object features can be estimated by the Euclidean distance between $X_{e,\mathrm{vision}}^{t}$ from the visual information and ${\hat{Z}}_{i}^{t}$. After the robot moves near the operation object, visual occlusion occurs. Therefore, the probability of the visual information for each particle sampled is defined as:

(10) $$p\left(X_{e,\mathrm{vision}}^{t}\left|{\hat{z}}_{i}^{t}\right.\right)=\left\{\begin{array}{cc} N\left({\hat{z}}_{i}^{t}\left|X_{e,\mathrm{vision}}^{t}\right.,\sigma_{v}^{2}\right), & d_{r}{>d}_{v}\\ 0, & d_{r}\leq d_{v} \end{array}\right.$$

where $\sigma_{v}^{2}$ is the covariance matrix of the Gaussian distribution function, $d_{r}$ is the distance between the robot and the target position of the uncertain object at the previous moment, $d_{v}$ is the minimum distance between the robot and the uncertain object without occlusion.

#### 3.1.6. Weighted Integration

To improve the accuracy of the estimation, the posterior probabilities obtained from these two information sources are integrated to obtain the weighting coefficient of the particles. At a certain moment, given the end position and robotic contact force, the weight of the particle can be expressed as:

(11) $$p\left({\hat{z}}_{i}^{t}\left|X_{\mathrm{end}}^{t},F_{e}{}^{t},X_{e,\mathrm{vision}}^{t}\right.\right)\propto \frac{p\left({\hat{z}}_{i}^{t}\left|X_{\mathrm{end}}^{t}\right.\right)\cdot p\left({\hat{z}}_{i}^{t}\left|X_{\mathrm{end}}^{t},F_{e}{}^{t}\right.\right)+p\left(X_{e,\mathrm{vision}}^{t}\left|{\hat{z}}_{i}^{t}\right.\right)}{L_{m}}p\left({\hat{z}}_{i}^{t}\right)=w_{i}^{t}\cdot p\left({\hat{z}}_{i}^{t}\right)$$

where $L_{m}$ is the normalization factor.

Redefine the Gaussian distribution of particles as:

(12) $$P=N(\mu_{z}^{t},\Sigma_{z}^{t})$$

where $\mu_{z}^{t}$ is the mean vector and $\Sigma_{z}^{t}$ is the covariance matrix.

(13) $$\mu_{z0}^{t}=\sum_{i=1}^{N}w_{i}^{t}{\hat{z}}_{i}^{t}$$

(14) $$\Sigma_{z}^{t}=AA^{T}$$

(15) $$A=\left[{\left(w_{i}^{t}\right)}^{\frac{1}{2}}\left({\hat{z}}_{i}^{t}-\mu_{z}^{t}\right),\cdots ,{\left(w_{N}^{t}\right)}^{\frac{1}{2}}\left({\hat{z}}_{N}^{t}-\mu_{z}^{t}\right)\right]$$

where $\mu_{z0}^{t}$ is features estimated starting value ${\hat{X}}_{d0}^{t}$ of the uncertain object.

#### 3.1.7. Correcting the Estimated Features of the Uncertain Object

**Figure 5:**
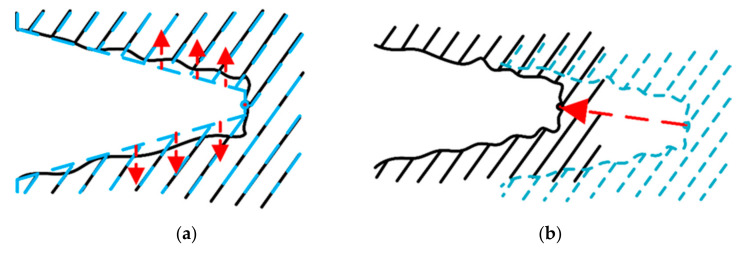
Schematic diagram of correction method. (**a**) Schematic diagram of memory adjustment correction method (**left**) and (**b**) schematic diagram of Inertia thinking correction method (**right**).

Since there may be errors in the shape and position of the uncertain object after sampling and estimation, historical information and rules of inertia thinking are used to correct the estimated value of the features of the uncertain object to improve the accuracy of the estimation, as shown in Figure 5. In this section, the memory adjustment correction method (MAC) and the Inertia thinking correction method (ITC) will be introduced in detail.

(1) Memory adjustment correction method

**Figure 6:**
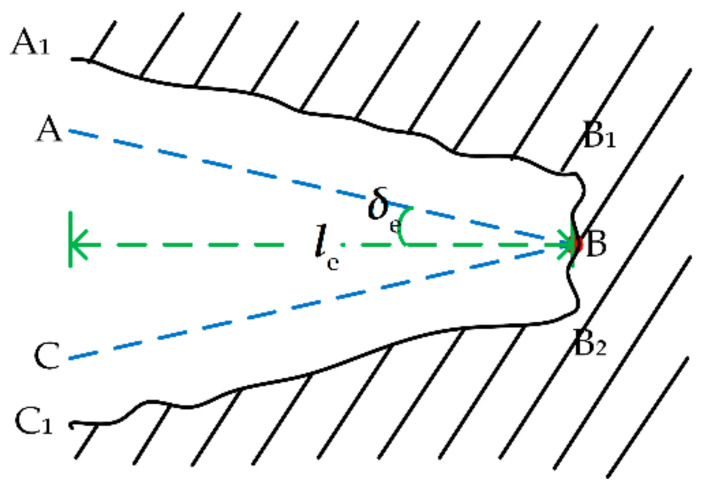
Shape features of the uncertain object.

The shape features of the uncertain object are calculated based on the estimated value, after completing the sampling estimation. According to the uncertain object proposed in Section 2, the shape is defined as the angle and the depth of the triangle, as shown in Figure 6. It is an accepted fact that dynamic effects do not change the shape of the uncertain object. Referring to the iterative update process of memory and operation during continuous human exploration, the fuzzy Naive Bayes principle is used to process real-time estimation and historical information.

According to the estimated value ${\hat{X}}_{d}$, the angle $\delta_{e,i}$ is calculated,

(16) $$\delta_{e1}=tan^{-1}\left[\left(P_{2}^{b}-P_{2}^{a}\right)/\left(P_{1}^{b}-P_{1}^{a}\right)\right]$$

(17) $$\delta_{e2}=tan^{-1}\left[\left(P_{2}^{b}-P_{2}^{c}\right)/\left(P_{1}^{b}-P_{1}^{c}\right)\right]$$

(18) $$\delta_{e}=\left[\begin{array}{cc} \delta_{e1} & \delta_{e2} \end{array}\right]$$

where $\delta_{e}$ is the angle vector. $P_{n}^{b}$ denotes the *n*-th element in $P_{}^{b}$. In the later sections, similar representations are used for this purpose.

To obtain the most suitable correction angle, historical information is introduced to participate in the correction calculation. The history information collection of angle is defined as $H_{\delta }=\left\{H_{\delta ,1},H_{\delta ,2},\cdots H_{\delta ,t-1}\right\}$, and the reference angle $\delta_{r}$ is calculated through the history information collection $H_{\delta }$.

The angle error $e_{\delta }$ is expressed as:

(19) $$e_{\delta ,i}=\delta_{r}-\delta_{e,i}$$

The posterior probability of the angle based on the angle error $\delta_{e}$ is defined as:

(20) $$p_{i}^{j}\left(\delta_{e,i}\left|e_{\delta ,i}\right.\right)=\mu_{i}^{}\left(e_{\delta ,i}\right)=\max\left(\mu_{i}^{j}\left(e_{\delta ,i}\right)\right),\left(j=1,2\right)$$

where $\mu_{i}^{j}\left(e_{\delta ,i}\right)$ is the fuzzy membership function, j is the labeled value, 1 is true and 2 is false.

A simple fuzzy system with two fuzzy rules is established, and the fuzzy membership function is:

(21) $$\mu_{i}^{1}\left(e_{\delta ,i}\right)=\left\{\begin{array}{cc} 1, & e_{\delta ,i}\leq \nu_{\delta ,1}\\ \frac{e_{\delta ,i}-\nu_{\delta ,2}}{\nu_{\delta ,1}-\nu_{\delta ,2}}, & \nu_{\delta ,1}<e_{\delta ,i}\leq \nu_{\delta ,2}\\ 0, & e_{\delta ,i}>\nu_{\delta ,2} \end{array}\right.$$

(22) $$\mu_{i}^{2}\left(e_{\delta ,i}\right)=\left\{\begin{array}{cc} 0, & e_{\delta ,i}\leq \nu_{\delta ,1}\\ \frac{e_{\delta ,i}-\nu_{\delta ,1}}{\nu_{\delta ,2}-\nu_{\delta ,1}}, & \nu_{\delta ,1}<e_{\delta ,i}\leq \nu_{\delta ,2}\\ 1 & e_{\delta ,i}>\nu_{\delta ,2} \end{array}\right.$$

where $\nu_{\delta ,1}$ and $\nu_{\delta ,2}$ are the membership function parameters.

$\delta_{\mathrm{cor}}$ is introduced to express the correction angle. When j is 11 (i.e., the labels corresponding to both $\delta_{e1}$ and $\delta_{e2}$ are 1), $\delta_{\mathrm{cor}}$ is $\delta_{e}$ corresponding to the maximum value of $p_{i}^{j=1}\left(\delta_{e,i}\left|e_{\delta ,i}\right.\right)$, and the credibility is recorded as the maximum value of $p_{i}^{j=1}\left(\delta_{e,i}\left|e_{\delta ,i}\right.\right)$. When j is 12 (i.e., only the label corresponding to $\delta_{e1}$ is 1), $\delta_{\mathrm{cor}}$ is $\delta_{e1}$, and the credibility is recorded as $p_{i=1}^{j=1}\left(\delta_{e,i}\left|e_{\delta ,i}\right.\right)$. When j is 21 (i.e., only the label corresponding to $\delta_{e2}$ is 1), $\delta_{\mathrm{cor}}$ is $\delta_{e2}$, and the credibility is recorded as $p_{i=2}^{j=1}\left(\delta_{e,i}\left|e_{\delta ,i}\right.\right)$. When j is 22 (i.e., the labels corresponding to both $\delta_{e1}$ and $\delta_{e2}$ are 2), $\delta_{\mathrm{cor}}$ is $\delta_{r}$, and the credibility is recorded as the maximum value of $p\left(•\right)=0.9$.

The history information at the current moment is defined as $H_{\delta ,t}={\left[\delta_{\mathrm{cor}},p\left(•\right)\right]}^{T}$. The historical information collection is regenerated as $H_{\delta }\leftarrow H_{\delta }\cup \left\{H_{\delta ,t}\right\}$. The reference angle $\delta_{r}$ is expressed as:

(23) $$\delta_{r}=\sum_{t=0}^{t=t-1}\frac{H_{\delta ,t,1}\cdot H_{\delta ,t,2}}{\sum H_{\delta ,t,2}}$$

The depth $l_{e,i}$ is calculated based on the estimated value ${\hat{X}}_{d0}$,

(24) $$l_{e1}=\left|P_{1}^{b}-P_{1}^{a}\right|$$

(25) $$l_{e2}=\left|P_{1}^{b}-P_{1}^{c}\right|$$

(26) $$l_{e}=\left[\begin{array}{cc} l_{e1} & l_{e2} \end{array}\right]$$

where $l_{e}$ is the depth vector.

In order to obtain the most suitable correction depth, historical information is introduced to participate in the correction calculation. The history information collection of depth is defined as $H_{l}=\left\{H_{l,1},H_{l,2},\cdots H_{l,t-1}\right\}$, and the reference depth $l_{r}$ is calculated through the history information collection $H_{l}$.

The depth error $e_{l}$ is expressed as:

(27) $$e_{l,i}=l_{r}-l_{e,i}$$

The posterior probability of the depth based on the depth error $l_{e}$ is defined as:

(28) $$p_{i}^{j}\left(l_{e,i}\left|e_{l,i}\right.\right)=\mu_{i}^{}\left(e_{l,i}\right)=\max\left(\mu_{i}^{j}\left(e_{l,i}\right)\right),\left(j=1,2\right)$$

where $\mu_{i}^{j}\left(e_{l,i}\right)$ is the fuzzy membership function, j is the labeled value, 1 is true and 2 is false. The calculation method and the same calculation method described above are omitted.

$l_{\mathrm{cor}}$ is introduced to express the correction depth. When j is 11 (i.e., the labels corresponding to both $l_{e1}$ and $l_{e2}$ are 1), $l_{\mathrm{cor}}$ is $l_{e,i}$ corresponding to the maximum value of $p_{i}^{j=1}\left(l_{e,i}\left|e_{l,i}\right.\right)$, and the credibility is recorded as the maximum value of $p_{i}^{j=1}\left(l_{e,i}\left|e_{l,i}\right.\right)$. When j is 12 (i.e., only the label corresponding to $l_{e1}$ is 1), $l_{\mathrm{cor}}$ is $l_{e1}$, and the credibility is recorded as $p_{i=1}^{j=1}\left(l_{e,i}\left|e_{l,i}\right.\right)$. When j is 21 (i.e., only the label corresponding to $l_{e2}$ is 1), $l_{\mathrm{cor}}$ is $l_{e2}$, and the credibility is recorded as $p_{i=2}^{j=1}\left(l_{e,i}\left|e_{l,i}\right.\right)$. When j is 22 (i.e., the labels corresponding to both $l_{e1}$ and $l_{e2}$ are 2), $l_{\mathrm{cor}}$ is $l_{r}$, and the credibility is recorded as the maximum value of $p\left(•\right)=0.9$.

The history information at the current moment is defined as $H_{l,t}={\left[l_{\mathrm{cor}},p\left(•\right)\right]}^{T}$. The historical information collection is regenerated as $H_{l}\leftarrow H_{l}\cup \left\{H_{l,t}\right\}$. The reference depth $l_{r}$ is expressed as:

(29) $$l_{r}=\sum_{t=0}^{t=t-1}\frac{H_{l,t,1}\cdot H_{l,t,2}}{\sum H_{l,t,2}}$$

After obtaining the correction angle and correction depth, the initial correction value ${\hat{X}}_{{}_{d,\mathrm{cor}}}^{t}$ of the features of the uncertain object is calculated:

(30) $$P_{\mathrm{cor},1}^{a}=P_{1}^{b}-l_{\mathrm{cor}}$$

(31) $$P_{\mathrm{cor},2}^{a}=\tan\left(\delta_{\mathrm{cor}}\right)\cdot l_{\mathrm{cor}}+P_{2}^{b}$$

(32) $$P_{\mathrm{cor}}^{b}=P_{}^{b}$$

(33) $$P_{\mathrm{cor},1}^{c}=P_{1}^{b}-l_{\mathrm{cor}}$$

(34) $$P_{\mathrm{cor},2}^{c}=-\tan\left(\delta_{\mathrm{cor}}\right)\cdot l_{\mathrm{cor}}+P_{2}^{c}$$

(35) $${\hat{X}}_{{}_{d,\mathrm{cor}}}^{t}=\left[\begin{array}{ccc} P_{\mathrm{cor}}^{a} & P_{\mathrm{cor}}^{b} & P_{\mathrm{cor}}^{c} \end{array}\right]$$

(2) Inertia thinking correction method

Human tactile perception is an iterative process of recognition–decision–correction. In this part, the features of the uncertain object will be corrected based on the result of the fused decision and the sensor information. For the sake of correcting the estimated features of the uncertain object, a novel method is proposed based on inertial thinking in the following.

Rule 1: When the robot moves towards the target position, the end of the robot should gradually approach the target position,

(36) $$\left\{\begin{array}{l} \gamma =\left({\hat{X}}_{d,\mathrm{cor},3}^{t}-X_{\mathrm{end},1}^{t}\right)-\left({\hat{X}}_{d,3}^{t-1}-X_{\mathrm{end},1}^{t}\right)\leq 0\\ {\dot{X}}_{\mathrm{end},1}^{t}\geq 0 \end{array}\right.$$

If $\gamma \geq 0\cup {\dot{X}}_{\mathrm{end},1}^{t}\geq 0$, it is necessary to correct the target position of the uncertain object based on rules of inertial thinking and the estimated target position at the current moment should be the target position at the previous moment, that is ${\hat{X}}_{d,\mathrm{cor}2,3}^{t}={\hat{X}}_{d,3}^{t-1}$. Otherwise, there is no need to correct again, that is, ${\hat{X}}_{d,\mathrm{cor}2,3}^{t}={\hat{X}}_{d,\mathrm{cor},3}^{t}$. However, when the end of the robot reaches the target position but still has not completed the task (Equation (51), the judgment method is given in the next section), the estimated target position of the uncertain object will shift toward the forward direction of the robot, that is, add a positive parameter.

(37) $$\left\{\begin{array}{l} \mathrm{unfinish}\\ e_{\mathrm{end},1}^{t}\leq \varsigma \end{array}\right.$$

where $e_{\mathrm{end}}^{t}$ is the distance between the end position of the robot and the estimated target position. ς is the threshold.

In the process of exploration, if humans perceive a collision, they will explore in the opposite direction.

Rule 2: If the end of the robot collides with $\vec{AB}$, the robot is expected to move away from $\vec{AB}$; if the end of the robot collides with $\vec{CD}$, the robot is expected to move away from $\vec{CD}$.

The end of the robot collides with $\vec{AB}$,

(38) $$\left\{\begin{array}{ll} P_{\mathrm{cor}2,2}^{b}=\tan\left(\delta_{\mathrm{cor}}\right)\cdot \left({\hat{X}}_{d,\mathrm{cor},3}^{t}-{\hat{X}}_{d,3}^{t-1}\right)+P_{\mathrm{cor},2}^{b}-\varepsilon , & {\hat{X}}_{d,\mathrm{cor},3}^{t}<{\hat{X}}_{d,3}^{t-1}\\ P_{\mathrm{cor}2,2}^{b}=-\tan\left(\delta_{\mathrm{cor}}\right)\cdot \left({\hat{X}}_{d,\mathrm{cor},3}^{t}-{\hat{X}}_{d,3}^{t-1}\right)+P_{\mathrm{cor},2}^{b}, & {\hat{X}}_{d,\mathrm{cor},3}^{t}\geq {\hat{X}}_{d,3}^{t-1} \end{array}\right.$$

The end of the robot collides with $\vec{CD}$,

(39) $$\left\{\begin{array}{ll} P_{\mathrm{cor}2,2}^{b}=-\tan\left(\delta_{\mathrm{cor}}\right)\cdot \left({\hat{X}}_{d,\mathrm{cor},3}^{t}-{\hat{X}}_{d,3}^{t-1}\right)+P_{\mathrm{cor},2}^{b}+\varepsilon , & {\hat{X}}_{d,\mathrm{cor},3}^{t}>{\hat{X}}_{d,3}^{t-1}\\ P_{\mathrm{cor}2,2}^{b}=\tan\left(\delta_{\mathrm{cor}}\right)\cdot \left({\hat{X}}_{d,\mathrm{cor},3}^{t}-{\hat{X}}_{d,3}^{t-1}\right)+P_{\mathrm{cor},2}^{b}, & {\hat{X}}_{d,\mathrm{cor},3}^{t}\leq {\hat{X}}_{d,3}^{t-1} \end{array}\right.$$

where ε is a constant positive parameter added to strictly ensure that the requirements of Rule 2 are met.

(40) $$P_{\mathrm{cor}2,1}^{a}=P_{\mathrm{cor}2,1}^{b}-l_{\mathrm{cor}}$$

(41) $$P_{\mathrm{cor}2,2}^{a}=\tan\left(\delta_{\mathrm{cor}}\right)\cdot l_{\mathrm{cor}}+P_{\mathrm{cor}2,2}^{b}$$

(42) $$P_{\mathrm{cor}2,1}^{c}=P_{\mathrm{cor}2,1}^{b}-l_{\mathrm{cor}}$$

(43) $$P_{\mathrm{cor}2,2}^{c}=-\tan\left(\delta_{\mathrm{cor}}\right)\cdot l_{\mathrm{cor}}+P_{\mathrm{cor}2,2}^{c}$$

(44) $$\mu_{z}^{t}={\hat{X}}_{d,\mathrm{cor}2}^{t}=\left[\begin{array}{ccc} P_{\mathrm{cor}2}^{a} & P_{\mathrm{cor}2}^{b} & P_{\mathrm{cor}2}^{c} \end{array}\right]$$

(45) $$X_{e}^{t}=P_{\mathrm{cor}2}^{b}$$

After the correction is completed, $\mu_{z}^{t}$ and $X_{e}^{t}$ are sent to the multi-sensor perception strategy part and the robot, respectively, $\mu_{z}^{t}$ is used to estimate the features of the uncertain object next time and $X_{e}^{t}$ is used to update the desired target position of the control section.

### 3.2. Fusion Decision Based on D-S Theory

**Figure 7:**
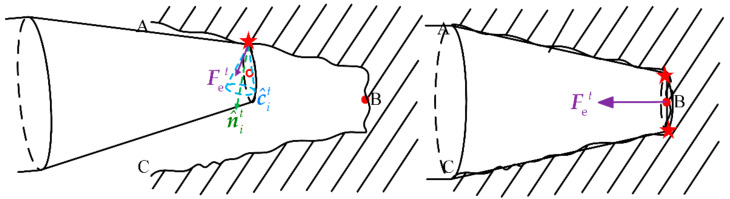
The force of the robot generated in different task progress stages.

As shown in Figure 7, the information of position and force is used to jointly decide the subsequent operation of the robot, based on the different information of position and force generated in different task progress stages. For example, the contact force during the task is lower than the one after the task is completed in general. In addition, the direction of the contact force is also different. The contact force during the task should be directed to the internal friction of the cone ${\hat{c}}_{i}^{t}$, while the task is completed on the contrary. Ideally, the direction of the contact force should coincide with the end direction.

The D-S theory is used to analyze the position information provided by the robot and the force information provided by the force sensor. This fusion decision strategy provides a mechanism to represent and process the uncertainty from robots and force sensors. Moreover, Dempster’s combination rules [26] are used to fuse information from different sources. 

First, the recognition framework Θ is defined as:

(46) $$\Theta =\left\{\mathrm{Finish},\mathrm{Unfinish}\right\}$$

The main elements of the recognition framework, $2^{\Theta }$, are defined as:

(47) $$\Omega =\left\{\left\{\mathrm{Finish}\right\},\left\{\mathrm{Unfinish}\right\},\left\{\mathrm{Finish},\mathrm{Unfinish}\right\}\right\}$$

where $\left\{\mathrm{Finish},\mathrm{Unfinish}\right\}$ represents the uncertain assumption in D-S theory. $\left\{\mathrm{Finish}\right\}$ and $\left\{\mathrm{Unfinish}\right\}$ represent the thresholds of the two hypotheses, respectively.

Next, the basic probability assignment (BPA) of different categories to which different information sources belong is calculated. In the method in this paper, the fuzzy naive Bayes method is used to generate BPA for each category and assign it to D-S theory. Let $V_{i}{}^{j}$ be the eigenvalue vector collected by each information source, where i represents the i-dimensional independent feature variable and j represents different information sources. For the position information source, $V_{i}$ is the position error in each direction. For the force information source, $V_{i}$ is the magnitude and direction of the contact force. $W\in C=\left\{C_{1},C_{2},\cdots ,C_{N}\right\}$ is defined as the classification label corresponding to $V_{i}$. In order to determine the BPA, the fuzzy naive Bayes method is used to determine the conditional probability and assign it to the basic probability used in the D-S theory,

(48) $$m\left(C_{i}\right)=\mu_{C_{i}}\left(V_{}{}^{j}\right)$$

where $C_{i}\in \left\{\left\{\mathrm{Finish}\right\},\left\{\mathrm{Unfinish}\right\}\right\}$.

According to D-S theory, there is a compound hypothesis that an object may belong to both $\left\{\mathrm{Finish}\right\}$ and $\left\{\mathrm{Unfinish}\right\}$. Therefore, the operator ∧ is used to assign the basic probability of $\left\{\mathrm{Finish},\mathrm{Unfinish}\right\}$,

(49) $$m\left(\left\{\mathrm{Finish},\mathrm{Unfinish}\right\}\right)=\mu_{\left\{\mathrm{Finish},\mathrm{Unfinish}\right\}}\left(V_{}{}^{j}\right)=\mu_{\left\{\mathrm{Finish}\right\}}\left(V_{}{}^{j}\right)\wedge \mu_{\left\{\mathrm{Unfinish}\right\}}\left(V_{}{}^{j}\right)$$

where ∧ is the minimum t-norm operation. Moreover, the purpose of normalizing the BPA solved above is to ensure the effectiveness of BPA,

(50) $$m\left(C_{i}\right)=\frac{\mu_{C_{i}}\left(V_{}{}^{j}\right)}{L}$$

(51) $$m\left(\left\{\mathrm{Finish},\mathrm{Unfinish}\right\}\right)=\frac{\mu_{\left\{\mathrm{Finish},\mathrm{Unfinish}\right\}}\left(V_{}{}^{j}\right)}{L}$$

(52) $$L=m\left(C_{i}\right)+m\left(\left\{\mathrm{Finish},\mathrm{Unfinish}\right\}\right)$$

where L is the normalization factor. BPA generated by different information sources can be obtained through the above methods. Then, Dempster’s combination rule is used to integrate the above BPA to obtain the overall BPA. Let $m_{1}$ and $m_{2}$ be the evidence provided by two independent information sources. In the framework of evidence theory, Dempster’s combination rule is expressed as $m=m_{1}⊕m_{2}$. The calculation method is as follows:

(53) $$m\left(A\right)=m_{1}⊕m_{2}\left(A\right)=\frac{1}{1-\kappa }m_{\wedge }\left(A\right)$$

where A∈Ω, A≠∅, $m_{\wedge }\left(A\right)$ represents the sum of BPA products whose intersection with the subset is not an empty set,

(54) $$m_{\wedge }\left(A\right)=\sum_{A_{1}\cap A_{2}=A}m_{1}\left(A_{1}\right)m_{2}\left(A_{2}\right)$$

where κ is the degree of conflict between evidence. The greater the degree of inconsistency between the information, the closer κ will be to 1. The sum of BPA products whose intersection is an empty set.

(55) $$\kappa =\sum_{A_{1}\cap A_{2}=\emptyset }m_{1}\left(A_{1}\right)m_{2}\left(A_{2}\right)$$

where 1−κ can be understood as a normalization factor.

For systems with multiple information sources, the overall BPA , $m_{\mathrm{all}}$ , can be expressed as:

(56) $$m_{\mathrm{all}}=m_{1}⊕m_{2}⊕\cdots ⊕m_{j}$$

After the fusion is completed, the entire decision-making process has changed from multiple information sources to single information source decision-making. Choose the hypothesis with the greatest probability as the predicted category of the sample in the test data. Finally, the result of the task assessment ξ and its BPA are obtained, where ξ is 0 or 1. 1 represents $\left\{\mathrm{Finish}\right\}$, 0 represents $\left\{\mathrm{Unfinish}\right\}$.

## 4. Design of Control System

### 4.1. Dynamics Model

The n-degree-of-freedom robot dynamics are:

(57) $$M\left(q\right)\ddot{q}+C\left(q,\dot{q}\right)\dot{q}+G\left(q\right)+D\left(q\right)+J^{T}F_{e}=\tau$$

where $M\left(q\right)\in R^{n\times n}$ is a symmetric positive definite inertia matrix, $C\left(q,\dot{q}\right)\in R^{n\times n}$ is the Coriolis force and centrifugal force matrix, $G\left(q\right)\in R^{n}$ is the gravity vector, $D\left(q\right)\in R^{n}$ is the friction torque matrix generated by the clearance. $q\in R^{n}$,$\dot{q}\in R^{n}$ and $\ddot{q}\in R^{n}$ is the position, velocity and acceleration of the robot in the joint space, respectively, obtained by the robot joint encoder. $J\in R^{n\times m}$ is the Jacobian matrix. $F_{e}\in R^{m}$ is the contact force vector at the end of the robot, which is collected by the six-dimensional force/torque sensor at the robotic end. $\tau \in R^{n}$ is the joint torque. It is worth noting that, $M\left(q\right)$, $C\left(q,\dot{q}\right)$ and $G\left(q\right)$ are all unknown.

The above formula is rewritten into Cartesian coordinate form as:

(58) $$M\left(q\right)J^{†}\left({\ddot{X}}_{\mathrm{end}}-\dot{J}\dot{q}\right)+C\left(q,\dot{q}\right)J^{†}{\dot{X}}_{\mathrm{end}}+G\left(q\right)+D\left(q\right)+J^{T}F_{e}=\tau$$

where $J^{†}=J^{T}{\left(JJ^{T}\right)}^{-1}$ is the pseudo-inverse matrix of the Jacobian matrix. $X_{\mathrm{end}}\in R^{m}$, ${\dot{X}}_{\mathrm{end}}\in R^{m}$ and ${\ddot{X}}_{\mathrm{end}}\in R^{m}$ are the end position, velocity and acceleration of the Cartesian space robot, respectively. It is particularly noted that the robot and the operating tool are considered as a whole and thus the robotic end is defined as the end of the operating tool.

Rewrite the above formula further:

(59) $$\bar{M}J^{†}\left({\ddot{X}}_{\mathrm{end}}+\Delta f\right)+J^{T}F_{e}=\tau$$

where $\bar{M}$ is the estimated value of the inertia matrix $M\left(q\right)$. Δf is uncertain terms, which can be expressed as:

(60) $$\Delta f=J{\bar{M}}^{†}\left(\left(M\left(q\right)-\bar{M}\right)J^{†}{\ddot{X}}_{\mathrm{end}}-M\left(q\right)\dot{J}\dot{q}+C\left(q,\dot{q}\right)J^{†}{\dot{X}}_{\mathrm{end}}+G\left(q\right)+D\left(q\right)\right)$$

### 4.2. Control System Designed with MSP

**Figure 8:**
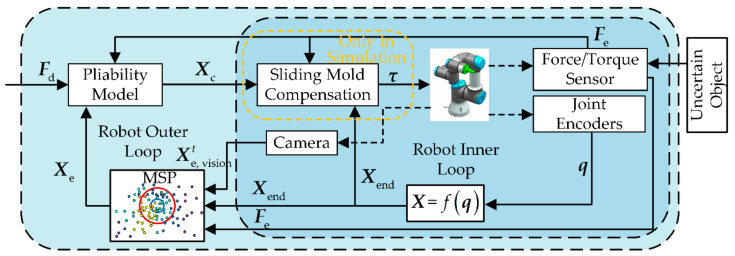
Control system designed with MSP.

In this part, the uncertain of the robot model and the uncertain of the object are considered in the design of the robot controller. In the outer loop, the pliability model is introduced and combined with a multi-sensor perception strategy to perceive the object and provide a command position $X_{c}$ for the inner loop. In the inner loop, sliding mode control is used to solve the influence of robot model errors (only used in simulation). The control system diagram is shown in Figure 8. It is worth noting that the sliding mode compensation term (yellow box in Figure 8) is only used in the simulation, while in the experiments the robot receives the command positions provided by the flexibility model directly. The sliding mode compensation term is introduced to reduce the impact of errors of the robot model established in Section 4.1 on the strategy validation.

The pliability model is:

(61) $$M_{d}\left({\ddot{X}}_{e}-{\ddot{X}}_{c}\right)+B_{d}\left({\dot{X}}_{e}-{\dot{X}}_{c}\right)+K_{d}\left(X_{e}-X_{c}\right)=F_{d}-F_{e}$$

where $M_{d}$, $B_{d}$ and $K_{d}$ are the inertia matrix, damping matrix and stiffness matrix required by the impedance model, respectively, and they are positive definite diagonal matrices. $X_{e}$ is the desired target position in Cartesian space, which is given by the perception strategy in the previous Section 3. ${\dot{X}}_{e}$ and ${\ddot{X}}_{e}$ are the desired velocity and acceleration in Cartesian space, respectively, which can be calculated by $X_{e}$. $F_{d}$ is the desired contact force. Rewrite the above formula:

(62) $$M_{d}{\ddot{X}}_{c}+B_{d}{\dot{X}}_{c}+K_{d}X_{c}=F_{e}-F_{d}+M_{d}{\ddot{X}}_{e}+B_{d}{\dot{X}}_{e}+K_{d}X_{e}$$

among them, $X_{e}\left(0\right)=X_{c}\left(0\right)$ and ${\dot{X}}_{e}\left(0\right)={\dot{X}}_{c}\left(0\right)$. The command position and command acceleration can be obtained from the command speed.

The position error is defined as:

(63) $$e=X_{c}-X_{\mathrm{end}}^{}$$

Sliding mode is defined as:

(64) $$S=\dot{e}+\lambda e$$

where λ is a positive definite constant matrix.

The robot reference state is defined as:

(65) $${\ddot{X}}_{r}={\ddot{X}}_{c}+\lambda \dot{e}$$

The controller can be designed as:

(66) τ=M¯J†X¨r+AS+ K ^signS+JTFe

## 5. Simulation and Experimental Results

Simulation and physical robot task experiments are designed for the narrow uncertain object. The proposed whole solution scheme is evaluated in terms of intelligence, autonomy and safety. (1) accuracy of interactive exploration for the features of the uncertain object $X_{d}$ (intelligence); (2) correctness of fusion decision for task completion judgments (autonomy) and (3) comparative experiments to assess the safety of the entire solution scheme for robotic autonomy operations (safety).

### 5.1. Simulation Studies

#### 5.1.1. Simulation Settings

Let n=4 in Equation (57). The complete simulation model is established based on the robot dynamics model and control system in Section 4. In the simulation, the basic parameters used in the control system are as follows.

Impedance coefficient:

$$M_{d}=\mathrm{diag}\left(\left[0.0000001,0.0000001\right]\right)$$

$$B_{d}=\mathrm{diag}\left(\left[5.05,5.05\right]\right)$$

$$K_{d}=\mathrm{diag}\left(\left[1500,1500\right]\right)$$

Sliding mode parameters:

(67) $$\lambda =\mathrm{diag}\left(\left[50000,50000\right]\right)$$

$$A=\mathrm{diag}\left(\left[20,20\right]\right)$$

(68) $$\hat{K}=7000$$

Inertial matrix estimates:

(69) $$\bar{M}=\mathrm{diag}\left(\left[0.005,0.0015,0.0015,0.01\right]\right)$$

In the simulation, the initial position of the robot is set near the operation object (i.e., there is visual occlusion). The initial features of the uncertain object are given and are in error with the expected value. Since the estimates of our method are based on the previous moment’s ones each time, the range of applicability of interactive exploration can be tested by adjusting the initial error. Considering that the entire operating object should be stabilized after the task is completed, the preload force is introduced and was set to 5 N.

#### 5.1.2. Intelligibility Evaluation

**Figure 9:**
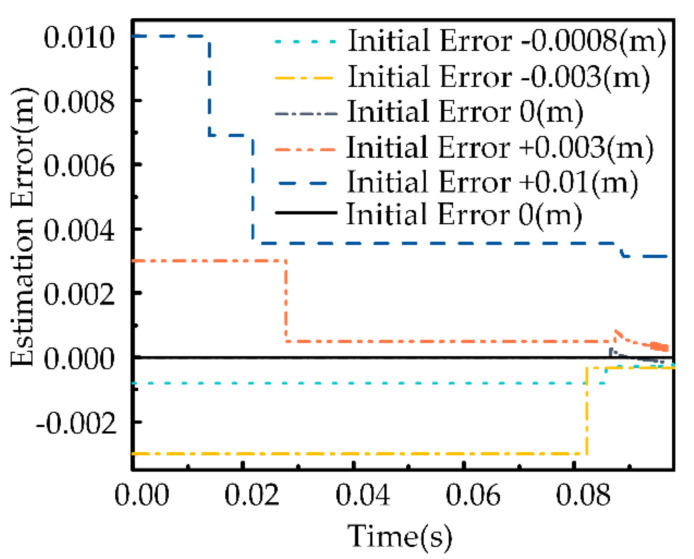
Estimation error of the target position of the uncertain object.

We give the initial features of the uncertain object, and the errors of these features (target position) from the expected values are −0.8 mm, −3 mm, 0, 3 mm and 10 mm, respectively. As can be seen from Figure 9, the accuracy of estimated values improves with the increasing number of explorations. The final error is less than 1mm. Therefore, it can be concluded that force-tactile exploration can estimate the target position of the uncertain object.

**Figure 10:**
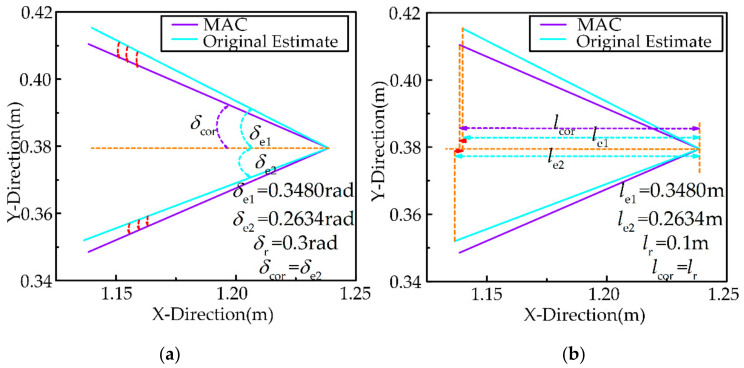
Simulation results of MAC. (**a**) simulation results of MAC (angle) and (**b**) simulation results of MAC (depth).

In addition, to verify the advantages of the proposed method in terms of estimation accuracy, we also evaluate the MAC and ITC methods. The simulation results of MAC are represented in Figure 10, where Figure 10a shows the simulation results of MAC (angle) and Figure 10b shows the simulation results of MAC (depth). Since features of the true object are uncertain, we consider the reference eigenvalues as the baseline. When one of the original estimated angle features is close to the reference angle, the original estimated angle feature is corrected to the closer one by MAC, as shown in Figure 10a. When none of the original estimated depth features is close to the reference eigenvalue, the original estimated depth feature is corrected for the reference depth by MAC, as shown in Figure 10b. We can arrive at a conclusion that the MAC method keeps the estimated shape features (depth and angle) of the uncertain object more accurately by historical data.

**Figure 11:**
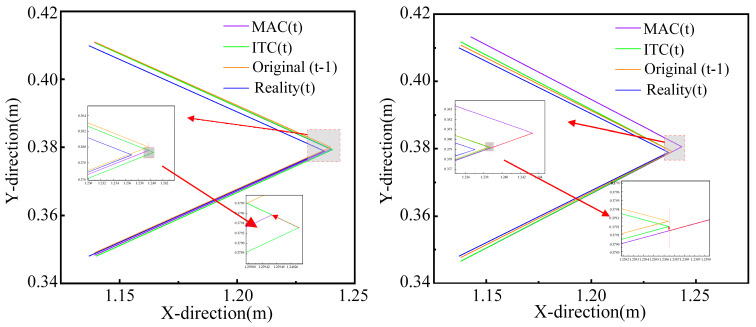
Simulation results of ITC. (**a**) result 1 and (**b**) result 2.

In Figure 11, the robot collides with BC at this time. In Figure 11a, the feature (target position) of the uncertain object at the current moment estimated by the MAC is located at the lower right of the estimated target position of the uncertain object at the last moment. After the ITC, the target position of the uncertain object, which is corrected by the MAC, at the current moment slides along the direction $\vec{BA}$ to near the inertial position. In Figure 11b, The target position feature of the uncertain object at the current moment estimated by the MAC is located at the upper right of the estimated target position of the uncertain object at the last moment. After the ITC, the target position of the uncertain object, which is corrected by the MAC, at the current moment slides along the direction $\vec{BC}$ to near the inertial target position, due to the presence of ε, which aims to make the robot conform to the collision-avoidance inertial response.

During the ITC design stage, we consider four possible scenarios that can cause inaccurate estimation (only three scenarios have appeared in the results of many simulations so far). Moreover, with the introduction of the ITC, the estimation results of the interactive exploration method are more accurate. Therefore, the validity of the design in this paper was verified.

#### 5.1.3. Autonomy Evaluation

**Figure 12:**
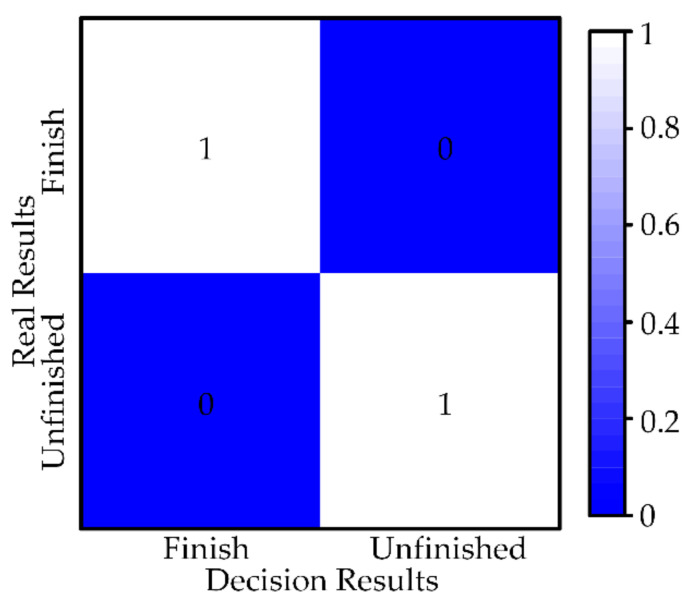
Confusion matrix for fusing decision results.

The fusion decision results can be viewed as a classification of the current task progress (finished and unfinished). Therefore, the confusion matrix is proposed to evaluate the performance of the fusion decision strategy. As shown in Figure 12, each row of the confusion matrix represents a real result (finished and unfinished) and each column represents the result of the fusion decision. Six simulation studies are selected for analysis, containing the results of 86 decisions. In 86 results, the number of finished is 6 and the number of unfinished is 80. The number on the box indicates the percentage of the result of all decisions. Since each task can have only one result of finished, we count the results of 86 decisions and calculate the percentages according to completion and unfinished, respectively, to represent all results in the range [0, 1]. As expected, the diagonal values of the confusion matrix are high, which indicates that the strategy has a high truth rate.

#### 5.1.4. Safety Evaluation

**Figure 13:**
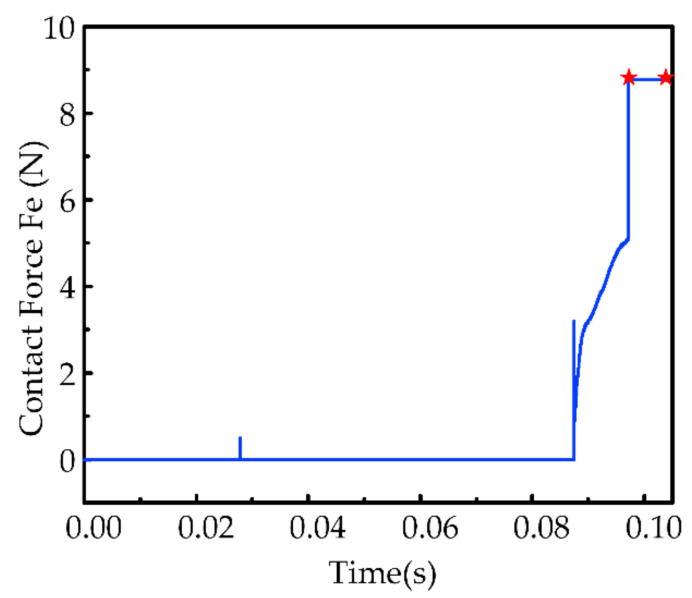
Contact force at the end of the robot.

Figure 13 shows that the contact force between the robot and the object under the control of the designed solution scheme is less than 10N, which satisfies the requirement of pliability and safety. In addition, the preload force of the robot in completing the task meets the design requirements (red marker), which proves that the task is qualified. In overview, the control system meets the requirements for contact forces presented in Section 2.

**Figure 14:**
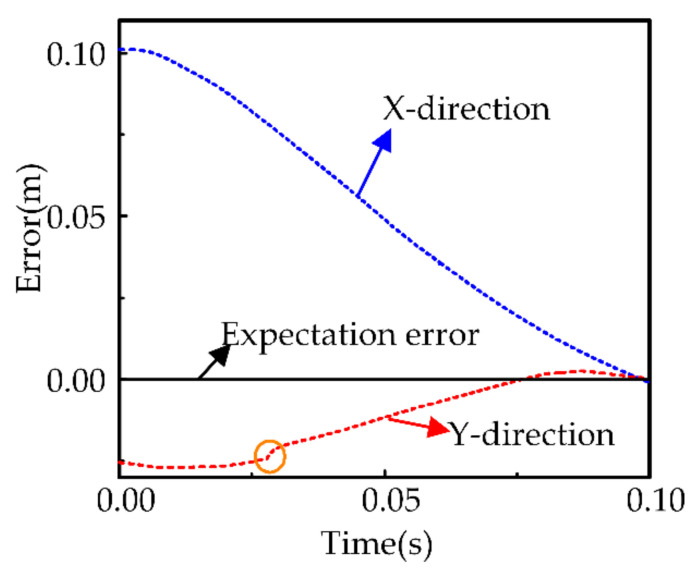
Robot position tracking error.

The result in Figure 14 shows that the proposed system can control the robot to reach the target position of the task with an error of less than 0.5 mm. We can also observe that the robot undergoes an abrupt displacement (represented as a circle in the y-direction), which is caused by the large change in the target position of the uncertain object estimated twice. Sudden displacement is within 1 mm due to the combined efforts of fusion decision results and impedance control. The later motion trajectory is smoother because the change in position between two adjacent estimates became smaller.

### 5.2. Experimental Studies

#### 5.2.1. Experimental Settings

**Figure 15:**
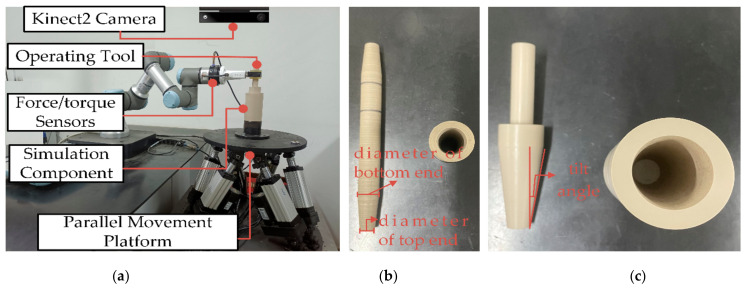
Experimental equipment. (**a**) Experimental equipment, (**b**) Operation tool 1 and operation object 1, (**c**) Operation tool 2 and operation object 2.

To further validate the performance of the proposed strategy in more complex and uncertain objects, a peg-in-hole experiment with dynamic effects is designed, and the experimental equipment is shown in Figure 15a. The experimental system consists of the UR3 robot, operating tools, simulation components (operating objects), a six-degree-of-freedom parallel movement platform, and a console. Among them, the six-degree-of-freedom parallel movement platform, which introduces uncertainty for the operating object, is used to increase the difficulty of the task position. The simulation component is fixed on the movement platform. The amplitude and frequency of the movement platform are set according to the simulation parameters and results. An omnidirectional depth camera Kinect2 is utilized to collect the features of the simulated component. The experimental code is written in python. The conversion of the coordinate system between each experimental equipment was determined before the experiment and unify with the model. The movement platform parameters are set as follows, $x=Asin\left(\;,2\pi ft\right)$, A=1~3 mm, f=1~5 Hz. The direction of motion of the platform is x-direction. A total of 75 insertion experiments are tested in the experiment. The preload force is set to 5 N.

Two sets of workpieces (operating tools and simulation components) are used in the experiment to verify the versatility of the proposed method for different workpieces. As shown in Figure 15b,c, there is a greater dimensional difference in the tools and objects of workpiece 2 compared to workpiece 1. Their basic sizes are as follows.

Operation tool 1: full length is 176 mm, diameter of bottom end is 20 mm, diameter of top end is 40 mm, tilt angle $\delta_{e}$ is 0.12 rad.

Operation object 1: hole deep is 80 mm, hole diameter of bottom end is 20 mm, hole diameter of top end is 40 mm, and tilt angle $\delta_{e}$ is 0.12 rad.

Operation tool 2: full length is 197 mm, diameter of bottom end is 19 mm, diameter of top end is 13 mm, tilt angle $\delta_{e}$ is 0.1 rad.

Operation object 2: hole deep is 32 mm, hole diameter of bottom end is 13 mm, hole diameter of top end is 20 mm, and tilt angle $\delta_{e}$ is 0.11 rad.

In the experiment, the basic parameters used in the control system are as follows.

Impedance coefficient:

$$\begin{array}{c} M_{d}=\mathrm{diag}\left(\left[0.2,0.2,0.2\right]\right)\\ B_{d}=\mathrm{diag}\left(\left[101,101,101\right]\right)\\ K_{d}=\mathrm{diag}\left(\left[300,300\right]\right) \end{array}$$

**Figure 16:**
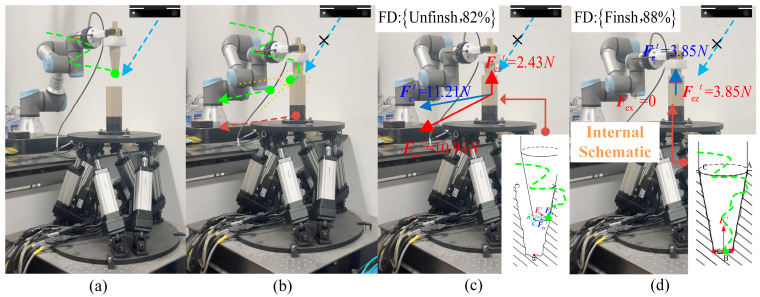
Snapshot of the robot peg-in-hole operation in the uncertain object with dynamic effects. (**a**) starting assembly without visual occlusion, (**b**) assemble with visual occlusion, (**c**) decision: Unfinish and (**d**) decision: Finish.

Figure 16 illustrates an example of a robot performing a peg-in-hole task in the uncertain object. The example shows the adaptation of the robotic motion during the dynamic effects of the uncertain object in operation and the update of the target position of the uncertain object. The green dashed line indicates the robotic operation trajectory. In Figure 16a, the perception strategy guides the robot to start the assembly. At this time, the visual information (blue dashed line) is weighted higher due to the non-existence of visual occlusion (the distance between the robot and the simulated component is greater than $d_{v}$). In Figure 16b, the perception strategy guides the robot to assemble inside the operating object. At this time, the tactile information is weighted higher while the visual information is weighted lower, due to the visual occlusion (the distance between the robot and the simulated component is less than $d_{v}$). The simulation component is in dynamic effects and the robot adjusted its motion accordingly, updating the target position by interactive exploration. In Figure 16c fusion decision is triggered and the result is Unfinish. In Figure 16d fusion decision is triggered and the result is Finish.

#### 5.2.2. Autonomy Evaluation

We first show the result of the fusion decision by the position and force on the end of the robot with the finished and unfinished operation, as shown in Figure 16c,d. Due to visual occlusion, the interior is not known. It is obvious that the right side operating tool has fully entered the simulation component from the outside, while the left side has only partially entered. From the collected position data, the distance between the robotic end position and the simulation component on the left side is 18mm, while the right side is less than 1mm. In addition, according to the data collected through the force/torque sensor, the direction of the contact force when the operation is finished is significantly different from that when the operation is unfinished. Combining the external observations with the collected data, we can find that the fusion decision turns out to be correct. It is indicated that the model we propose in the design of the fusion decision is correct. Moreover, the robot will continue to perform the task when the decision result is unfinished, and vice versa, it will stop the movement.

**Figure 17:**
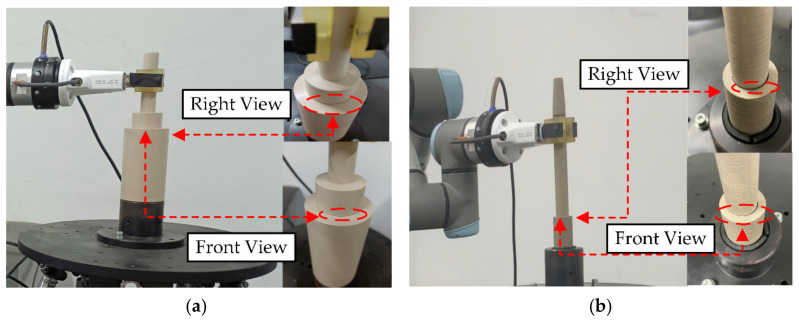
Detail at finished operation. (**a**) operation tool 1 and operation object 1, (**b**) operation tool 2 and operation object 2.

Some details are shown in Figure 17 to evaluate the quality of the operation. We observe from the front and side, respectively, that the operating tool is very tightly fitted to the simulation component (red circles). At the end of the experiment, we try to pull the operating tool out of the simulated component, which requires a force of approximately more than 10 N. It further indicates that the fusion decision is correct. Comparing Figure 17a,b, when using workpiece 2 for the experiment, there is a large clearance after the task is completed. It is caused by the difference in the size of operation tool 2 and operation object 2. Nevertheless, the proposed method can still guide the robot to complete the task. It also demonstrates the versatility of the method for different workpieces and reduces the workload of the operator which does not require the operator to modify the parameters after each workpiece change.

**Figure 18:**
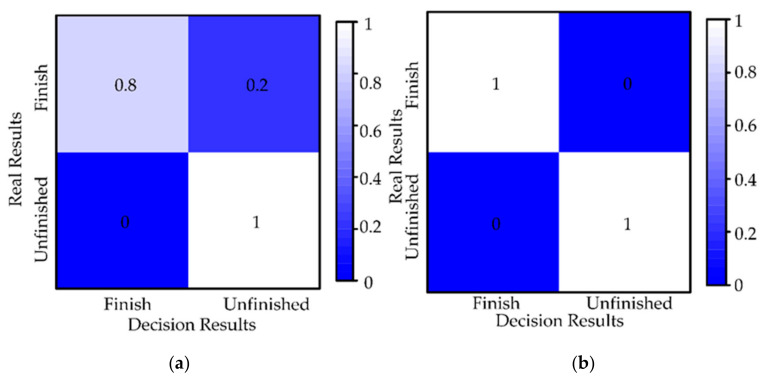
Confusion matrix for fusing decision results. (**a**) operation tool 1 and operation object 1, (**b**) operation tool 2 and operation object 2.

As shown in Figure 18, we count the results of 55 decisions and calculate the percentages according to finished and unfinished, respectively, to represent all results in the range [0, 1]. For the incorrect results, we find the reason for the decision error, one source of information with evidence showing a high probability of finished and the other showing a low probability of finished, which ultimately yields a low probability of finished. The problem can be solved by a threshold value of a higher probability of finished is set. The application of this strategy eliminates the need for the operator to check that the workpiece is securely mounted, which helps to reduce the workload.

#### 5.2.3. Safety Evaluation

**Figure 19:**
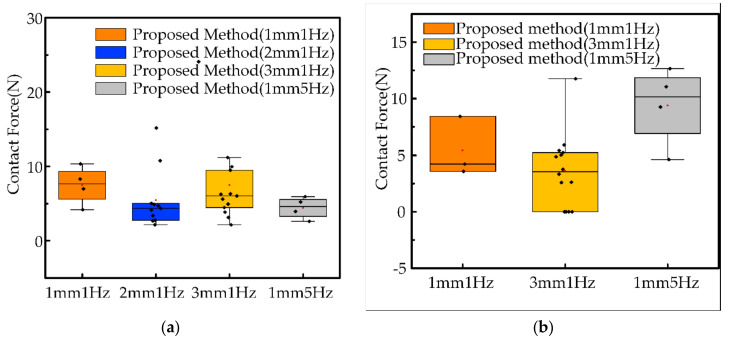
Results of operation of robots with different dynamic effects. (**a**) operation tool 1 and operation object 1, (**b**) operation tool 2 and operation object 2.

In this section, we conduct experiments on the proposed system under the dynamic effects conditions of A=1 mm & f=1 Hz, A=2 mm & f=1 Hz, A=3 mm & f=1 Hz and A=1 mm & f=5 Hz, as shown in Figure 19. In the experiments, we filter the contact force less than 2 N to counter the effect of measurement noise. The black dots in the figure represent the contact force generated by each collision, and the red dots represent the average value of the contact force for each experiment. As a whole, two workpieces of the mean contact forces are low, between 5 and 10 N, and the number of collisions is low, no more than 15 per experiment. It indicates that our solution scheme meets the requirement for safe operation for multiple types of dynamic effects. In Figure 19a, comparing orange, blue and yellow, we find that an increase in the amplitude of the dynamic effects causes a slight increase in the contact force, which also proves that the dynamic effects of the uncertain object affect the robot end operation.

**Figure 20:**
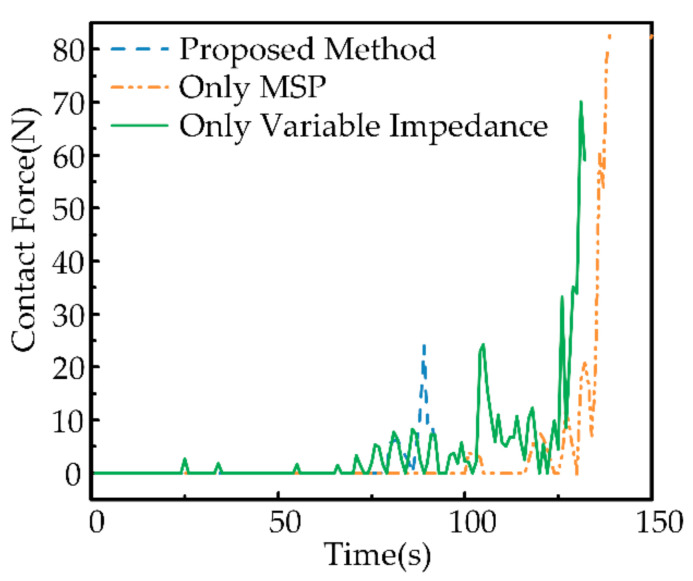
Results of operation of robots with different control methods.

In the previous experiment, we illustrated the generality of the proposed system in terms of safety. To verify the safety attributed to the proposed method, we compare the proposed system with two benchmark experiments: (1) guide operation by MSP only, without impedance control, and (2) variable impedance control only, without MSP. For each method, experiments are performed sequentially at three types of dynamic effects. As shown in Figure 20, we show the comparison results from the same dynamic effects, with one experiment for each method.

(1) The maximum contact force (orange) is demonstrated using only MSP-guided operation without impedance control. This result is expected and indicates that a pliability model is needed to accomplish the operation task to minimize the contact force between the robot and the uncertain object. Although the target position is updated by MSP, it leads to large contact forces when guiding the robot movement, which is undesirable since the robot–object interaction is not considered.

(2) Variable impedance control is a classical approach to deal with the problem of robot interaction with an uncertain object, which adjusts the impedance parameters online by contact forces to accommodate the uncertain object with dynamic effects. The approach using variable impedance control only without MSP (green) presents multiple peaks, which means that the robot guided only by variable impedance without MSP may lead to frequent collisions and possibly even divergence during operation. Frequent collisions can also exacerbate dynamic effects.

(3) In summary, the proposed method (blue) demonstrates minimal contact forces and no multiple collisions. It indicates that the method proposed in this paper is effective.

## 6. Conclusions

In this paper, we focused on the issue of robotic autonomy operations in the real-world unstructured environment. Missing or inaccurate visual information was also considered due to confined space limitations and interference from complex environments. In order to satisfy the three requirements of intelligence, autonomy and safety, a multi-sensor perception strategy for the robot was proposed to achieve a humanoid autonomy operation process integrating exploration, decision and guidance with uncertain objects. In terms of intelligence, it was our goal to obtain information about the features of the uncertain object. An interactive exploration method using Bayesian networks was proposed to integrate multimodal information and accurately estimate the features of the uncertain object, which can comprehensively perceive the features of the uncertain object even in the presence of visual occlusion. The exploration approach was general for static objects and multiple dynamic objects. In terms of safety, the proposed system was capable of performing tasks under the uncertain object and minimizing the forces of interaction between the robot and the uncertain object. In terms of autonomy, the proposed fusion decision strategy has enabled autonomous start–stop and guided subsequent operations of the robot, which could reduce the workload of the operators. Based on the D-S theory, the evidence information provided by multiple information sources was fused to judge the task progress, which gives the robot human-like decision-making capability. Moreover, the pliability model was combined with an MSP to reduce the interaction forces during operation. In general, the multi-sensor-based solution scheme showed fine performance for robotic operation tasks with both position and force requirements.

There is one more area where the proposed method could be improved. The inclusion of a pose adjustment strategy before the MSP will improve the generality of the method for pegs and holes with multiple angles. This attitude adjustment strategy performs a tilt-right–rotate–alignment process to bring the robotic end into an ideal attitude for operation.

## Data Availability

Not applicable.

## References

[1] Xie Z., Chen B., Liu J., Yuan F., Shao Z., Yang H., Domel A.G., Zhang J., Wen L. (2021). A Tapered Soft Robotic Oropharyngeal Swab for Throat Testing: A New Way to Collect Sputa Samples. IEEE Robot. Autom. Mag..

[2] Song R., Li F., Quan W., Yang X., Zhao J. (2021). Skill learning for robotic assembly based on visual perspectives and force sensing. Robot. Auton. Syst..

[3] Zhu W., Liu H., Ke Y. (2020). Sensor-Based Control Using an Image Point and Distance Features for Rivet-in-Hole Insertion. IEEE Trans. Ind. Electron..

[4] Jiang T., Cui H., Cheng X., Tian W. (2021). A Measurement Method for Robot Peg-in-Hole Prealignment Based on Combined Two-Level Visual Sensors. IEEE Trans. Instrum. Meas..

[5] Zou J. (2021). Predictive visual control framework of mobile robot for solving occlusion. Neurocomputing.

[6] Nagahama K., Yamazaki K. Learning from Demonstration Based on a Mechanism to Utilize an Object’s Invisibility. Proceedings of the 2019 IEEE/RSJ International Conference on Intelligent Robots and Systems (IROS).

[7] Kim D., Lee J., Chung W.-Y., Lee J. (2020). Artificial Intelligence-Based Optimal Grasping Control. Sensors.

[8] Kwiatkowski J., Lavertu J.-S., Gourrat C., Duchaine V., Okamura A.M., Amato N., Asfour T., Choi Y.J., Chong N.Y., Ding H., Lee D.H., Lerma C.C., Li J.S., Marchand E. (2019). Determining Object Properties from Tactile Events During Grasp Failure. Proceedings of the IEEE 15th International Conference on Automation Science and Engineering.

[9] Tian S., Ebert F., Jayaraman D., Mudigonda M., Finn C., Calandra R., Levine S. Manipulation by Feel: Touch-Based Control with Deep Predictive Models. Proceedings of the 2019 International Conference on Robotics and Automation (ICRA).

[10] Gomes D.F., Paoletti P., Luo S. (2021). Generation of GelSight Tactile Images for Sim2Real Learning. IEEE Robot. Autom. Lett..

[11] Geier A., Tucker R., Somlor S., Sawada H., Sugano S. (2020). End-to-End Tactile Feedback Loop: From Soft Sensor Skin Over Deep GRU-Autoencoders to Tactile Stimulation. IEEE Robot. Autom. Lett..

[12] Billard A., Kragic D. (2019). Trends and challenges in robot manipulation. Science.

[13] Bekiroglu Y., Detry R., Kragic D. Learning tactile characterizations of object- and pose-specific grasps. Proceedings of the 2011 IEEE/RSJ International Conference on Intelligent Robots and Systems.

[14] Calandra R., Owens A., Jayaraman D., Lin J., Yuan W., Malik J., Adelson E., Levine S. (2018). More Than a Feeling: Learning to Grasp and Regrasp Using Vision and Touch. IEEE Robot. Autom. Lett..

[15] Watkins-Valls D., Varley J., Allen P. Multi-Modal Geometric Learning for Grasping and Manipulation. Proceedings of the 2019 International Conference on Robotics and Automation (ICRA).

[16] Lv X., Chen G., Hu H., Lou Y. A Robotic Charging Scheme for Electric Vehicles Based on Monocular Vision and Force Perception. Proceedings of the 2019 IEEE International Conference on Robotics and Biomimetics (ROBIO).

[17] Jusoh S., Almajali S. (2020). A Systematic Review on Fusion Techniques and Approaches Used in Applications. IEEE Access.

[18] Lee M.A., Zhu Y., Zachares P., Tan M., Srinivasan K., Savarese S., Fei-Fei L., Garg A., Bohg J. (2020). Making Sense of Vision and Touch: Learning Multimodal Representations for Contact-Rich Tasks. IEEE Trans. Robot..

[19] Pastor F., García-González J., Gandarias J., Medina D., Closas P., Garcia A., Gomez-de-Gabriel J. (2020). Bayesian and Neural Inference on LSTM-Based Object Recognition from Tactile and Kinesthetic Information. IEEE Robot. Autom. Lett..

[20] Izatt G., Mirano G., Adelson E., Tedrake R. Tracking objects with point clouds from vision and touch. Proceedings of the 2017 IEEE International Conference on Robotics and Automation (ICRA).

[21] Zhang F., Cully A., Demiris Y. (2019). Probabilistic Real-Time User Posture Tracking for Personalized Robot-Assisted Dressing. IEEE Trans. Robot..

[22] Nottensteiner K., Sachtler A., Albu-Schäffer A. (2021). Towards Autonomous Robotic Assembly: Using Combined Visual and Tactile Sensing for Adaptive Task Execution. J. Intell. Robot. Syst..

[23] Sachtler A., Nottensteiner K., Kaßecker M., Albu-Schäffer A. Combined Visual and Touch-based Sensing for the Autonomous Registration of Objects with Circular Features. Proceedings of the 2019 19th International Conference on Advanced Robotics (ICAR).

[24] Thomas U., Molkenstruck S., Iser R., Wahl F.M. Multi Sensor Fusion in Robot Assembly Using Particle Filters. Proceedings of the Proceedings 2007 IEEE International Conference on Robotics and Automation.

[25] Liu L., Zhang J., Chen K., Wang H. (2014). Combined and interactive effects of interference fit and preloads on composite joints. Chin. J. Aeronaut..

[26] Liu Y.-T., Pal N.R., Marathe A.R., Wang Y.-K., Lin C.-T. (2017). Fuzzy Decision-Making Fuser (FDMF) for Integrating Human-Machine Autonomous (HMA) Systems with Adaptive Evidence Sources. Front. Neurosci..

